# Treatment cessation in HBeAg-negative chronic hepatitis B: clinical response is associated with increase in specific proinflammatory cytokines

**DOI:** 10.1038/s41598-023-50216-y

**Published:** 2023-12-18

**Authors:** Marte Holmberg, Hans Christian D. Aass, Olav Dalgard, Ellen Samuelsen, Dan Sun, Niklas K. Björkström, Asgeir Johannessen, Dag Henrik Reikvam

**Affiliations:** 1 Department of Infectious Diseases, Vestfold Hospital, Tønsberg, Norway; 2 Institute of Clinical Medicine, University of Oslo, Oslo, Norway; 3 Oslo University Hospital, Oslo, Norway; 4 Akershus University Hospital, Lørenskog, Norway; 5 Center for Infectious Medicine, Department of Medicine Huddinge, Karolinska Institutet, Karolinska University Hospital, Stockholm, Sweden

**Keywords:** Infectious diseases, Viral infection, Immunology, Chemokines, Cytokines

## Abstract

Patients with HBeAg-negative chronic hepatitis B may experience an immune response after stopping nucleos(t)ide analogue (NA)therapy, which may potentially trigger HBsAg loss or off-therapy sustained viral control. The immunological mechanisms determining clinical response remain poorly understood. To identify inflammatory signatures associated with defined outcomes, we analysed plasma cytokines and chemokines from 57 HBeAg-negative patients enrolled in the Nuc-Stop Study at baseline and 12 weeks after NA cessation. Clinical response at 12 weeks was classified into four groups: immune control, viral relapse, evolving clinical relapse, and resolving clinical relapse. Twelve weeks after treatment cessation 17 patients (30%) experienced immune control, 19 (33%) viral relapse, 6 (11%) evolving clinical relapse, and 15 (26%) resolving clinical relapse. There was a significant increase in interferon-γ-induced protein 10 (IP-10; p = 0.012) and tumor necrosis factor (TNF; p = 0.032) in patients with evolving clinical relapse. Sparse partial least-squares multivariate analyses (sPLS-DA) showed higher first component values for the clinical relapse group compared to the other groups, separation was driven mainly by IP-10, TNF, IL-9, IFN-γ, MIP-1β, and IL-12. Our results demonstrate that evolving clinical relapse after NA cessation is associated with a systemic increase in the proinflammatory cytokines IP-10 and TNF.

*Clinical trial registration*: ClinicalTrials.gov, Identifier: NCT03681132.

## Introduction

In 2016, the World Health Organization (WHO) launched a goal to eliminate viral hepatitis as a major public health threat by 2030^1^. Hepatitis B virus (HBV) infection is one of the leading causes of cirrhosis and hepatocellular carcinoma worldwide and is estimated to cause between 555,000 and 865,000 deaths per year^2–4^. There is no definite cure and suppressive nucleos(t)ide analogue (NA) therapy is the mainstay of treatment. Functional cure, defined as hepatitis B surface antigen (HBsAg) loss, is the ultimate treatment goal but is rarely achieved in patients on NA treatment, with an annual incidence of 0.15–0.33% in chronic hepatitis B (CHB) patients^5–8^. Treatment duration is therefore indefinite and potentially lifelong.

A decade ago, Hadziyannis et al. observed that 39% of patients lost HBsAg following NA therapy cessation in hepatitis B e antigen (HBeAg)-negative CHB patients. Since then, several studies have looked into whether stopping NA therapy can initiate an immune response that may trigger HBsAg loss^5,9–16^; however, the proportion who achieve this endpoint has been lower than what was reported in Hadziyannis’ study^5,7,11,15,17^. Identifying patients more likely to reach HBsAg loss is essential to avoid futile treatment stops. Furthermore, identifying patients who may experience detrimental outcomes of treatment cessation is crucial for patient safety.

The immune system plays a complex role in the course of HBV infection and gaining better knowledge of the immune mechanisms involved in the different phases of HBV infection is a vital step toward a treatment strategy to achieve functional cure. As signalling molecules of the immune system, cytokines have a fundamental role in both mediating and regulating the immune response. Rebound after treatment cessation in HBeAg-negative CHB has been reported to be associated with an increase in IFN-γ-induced protein 10 (IP-10), tumor necrosis factor (TNF), interleukin (IL)-10, and IL-12^14^. Cytokines and chemokines may have an unexploited potential as prediction biomarkers or therapeutic targets in CHB^18^. To attempt to identify the immunological mechanisms that determine clinical response and clinical relapse after treatment cessation, we analysed selected cytokines and chemokines at baseline and 12 weeks after treatment cessation in patients with HBeAg-negative CHB who participated in the Nuc-Stop Study^19^.

## Materials and methods

### Study design and participants

This was a sub-study of 57 CHB patients enrolled in the Nuc-Stop Study, a multicentre randomized trial where patients with HBeAg-negative CHB stopped NA therapy and were followed up for three years^19^. Inclusion took place between 2018 and 2020 from three centres in Norway: 26 from Oslo University Hospital, 25 from Akershus University Hospital, and 6 from Vestfold Hospital. All patients were non-cirrhotic and virally suppressed with tenofovir (including both tenofovir disoproxil fumarate and tenofovir alafenamide) or entecavir for at least 24 months prior to stopping NA therapy. None of the patients had coinfection with HIV, hepatitis C or hepatitis D, other active liver disease, previous hepatocellular carcinoma, or a history of decompensated liver disease. Patients with alcohol consumption of more than 14 (women) or 21 (men) standard units of alcohol per week during the past six months were excluded.

All study participants stopped NA therapy at inclusion and were followed up 4, 8, and 12 weeks after treatment cessation, and then 3-monthly until end-of-study. All study visits consisted of clinical evaluation and blood collection for analyses of HBV DNA and liver enzymes. In addition, serum and plasma at baseline and 12 weeks were stored at − 80 °C in a research biobank for later analyses. For the current analyses, plasma samples collected at baseline (the day of treatment withdrawal) and after 12 weeks were analysed.

### Multiplex cytokine assay

Plasma cytokines and chemokines were analysed on a Luminex IS 200 with the Bio-Plex Pro Human Cytokine 27-plex Assay (Cat. no.: M500KCAF0Y, Bio-Rad Laboratories Inc., Hercules, USA). The plasma samples were thawed and kept on ice, gently vortexed, and spun down at 10,000 g for 10 min at 4 °C. The supernatant of each sample was further diluted (1:4), and 50 µl was loaded into each well. Plasma from each individual donor was analysed on the same assay plate in duplicate. Internal kit controls were included to determine the intra- and inter-coefficient of variation expressed as a percentage (% CV). The plasma concentration of the following cytokines and chemokines was determined: IL-1β, IL-1 receptor agonist (RA), IL-2, IL-4, IL-5, IL-6, IL-7, IL-8, IL-9, IL-10, IL-12p70, IL-13, IL-15, IL-17, eotaxin, basic fibroblast growth factor (FGF), granulocyte-colony-stimulating factor (G-CSF), granulocyte–macrophage colony stimulating factor (GM-CSF), interferon (IFN)-γ, IP-10, monocyte chemoattractant protein (MCP)-1, macrophage-inflammatory protein (MIP)-1α, MIP-1β, platelet-derived growth factor (PDGF)-BB, regulated upon activation, normally T-expressed, and presumably secreted (RANTES), vascular endothelial growth factor (VEGF), and TNF. Any cytokine that was undetectable by the Luminex assay in more than 50% of the participants at either time point was excluded from subsequent statistical analyses. Consequently, IL-5, IL-10, IL-15, MIP-1α, and VEGF were omitted from further investigation.

### Definition of clinical response at 12 weeks

When analysing the change in cytokine concentrations from baseline to 12 weeks (delta value between baseline and 12 weeks), clinical response at 12 weeks was classified as follows:

- Immune control (HBV DNA ≤ 2000 IU/ml and alanine aminotransferase (ALT) ≤ 80 U/L)
- Viral relapse (HBV DNA > 2000 IU/ml and ALT ≤ 80 U/L)
- Evolving clinical relapse (HBV DNA > 2000 IU/ml, ALT > 80 U/L and increasing from week 8 to 12)
- Resolving clinical relapse (HBV DNA > 2000 IU/ml, ALT > 80 U/L and decreasing from week 8 to 12)

The rationale for choosing the above levels of differentiation for ALT and HBV DNA was based on the European Association for the Study of the Liver clinical practice guidelines^20^. ALT > 2 × ULN corresponds to ALT > 80 U/L.

**Figure 1:**
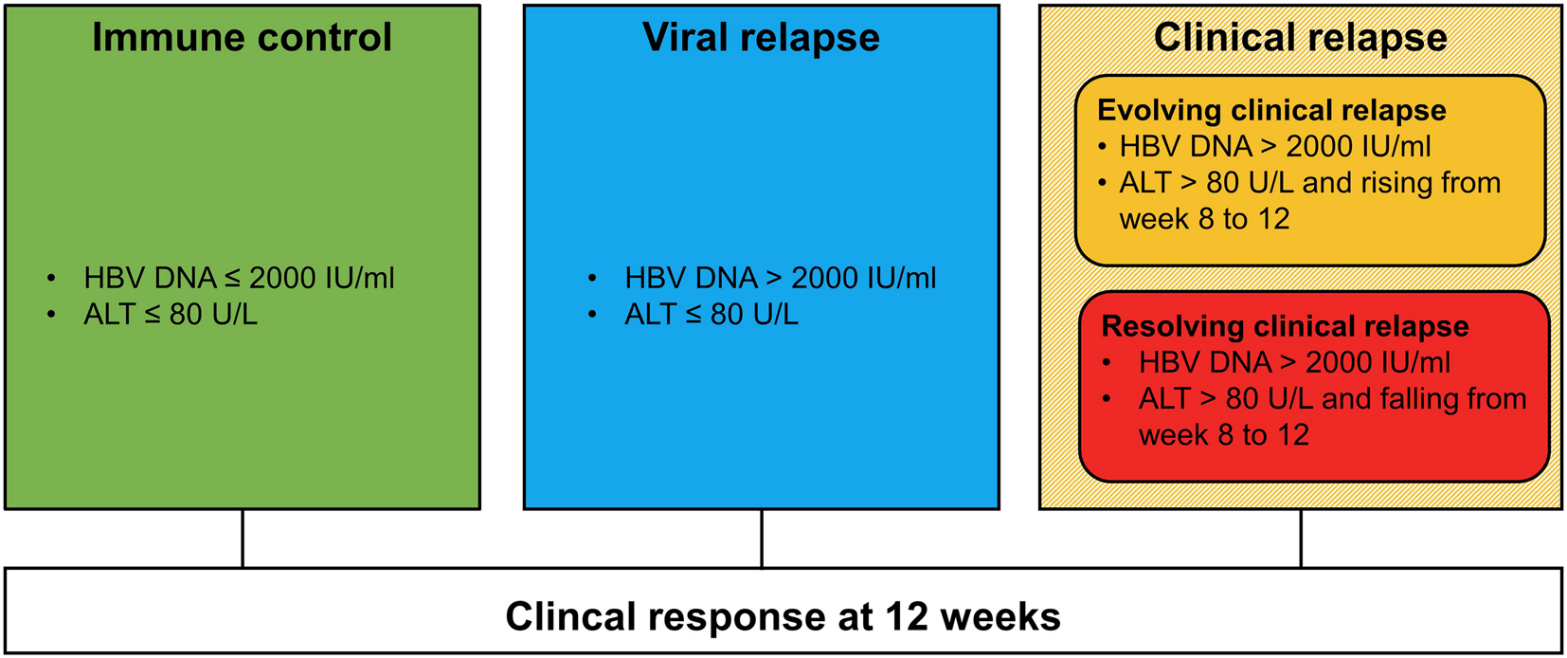
Clinical response at 12 weeks. The clinical relapse group was divided into evolving clinical relapse and resolving clinical relapse to better capture the difference between a progressing flare and a subsiding flare. *HBV* hepatitis B virus, *ALT* alanine aminotransferase.

Clinical relapse was divided into evolving clinical relapse and resolving clinical relapse based on whether ALT was increasing or decreasing from week eight to week twelve (Fig. 1). This was done to better capture the differences in immunological and inflammatory dynamics between a progressing flare and a subsiding flare.

Evolving clinical relapse and resolving clinical relapse were merged into one group (“clinical relapse”) for the analysis of the baseline concentrations of the cytokines, since the timing of relapse was then irrelevant.

### Statistical analyses

Comparisons between three or four groups of continuous variables were performed by Kruskal–Wallis test, followed by Bonferroni post hoc tests. Categorical variables were compared using Fisher´s exact test. In addition, a multivariate supervised sparse Partial Least Square Discriminant Analysis (sPLS-DA) was used to investigate differences in cytokine profiles at baseline and differences in change in cytokines from baseline to 12 weeks. Correlations between two continuous variables were analysed using Spearman´s test. To examine the predictive value of baseline variables, a receiver operating characteristic (ROC) analysis was performed. The area under the ROC curve (AUC), sensitivity, specificity, and predictive values were calculated. Statistical analyses were performed using IBM SPSS Statistics version 28 (SPSS Inc., Chicago, IL, USA) and STATA SE 17 (StataCorp, College Station, TX, USA). sPLS-DA was executed using mixOmics R package version 6.24.0^21^. P-values less than 0.05 were considered statistically significant.

### Ethical consideration

This study was conducted in compliance with the Declaration of Helsinki and International Conference on Harmonisation/Good Clinical Practice. The Regional Committees for Medical and Health Research Ethics (REK No. 2018/998) and the Norwegian Medicines Agency (EudraCTnr. 2018-000724-34) approved the protocol. The Nuc-Stop Study was registered at ClinicalTrials.gov (Identifier: NCT03681132). All patients signed informed consent to participate in the study.

### Ethics approval statement

This study was conducted in compliance with the Declaration of Helsinki and International Conference on Harmonisation/Good Clinical Practice. The Regional Committees for Medical and Health Research Ethics (REK No. 2018/998) and the Norwegian Medicines Agency (EudraCTnr. 2018-000724-34) approved the protocol.

### Patient consent statement

All patients signed informed consent to participate in the Nuc-Stop Study.

## Results

### Clinical characteristics of the study population

The baseline characteristics of the 57 study participants are shown in Table 1. The median age was 45 (interquartile range 40–52) years, and 72% were men. All the most common HBV genotypes were represented, and genotypes, age, sex, and ethnic background were evenly distributed among the four clinical response groups. All patients were treated with potent NAs with a high barrier to resistance at enrolment, with tenofovir as the most common treatment (74%).

**Table 1:** Baseline characteristics of the study participants, grouped by clinical and virological response at 12 weeks.

	Immune control	Viral relapse	Evolving clinical relapse	Resolving clinical relapse	P value
N (%)^a^	17 (30)	19 (33)	6 (11)	15 (26)	NA
Age (years)^b^	45 (41–54)	43 (40–51)	47 (41–51)	48 (39–58)	0.791^c^
Men^a^	14 (82)	11 (58)	4 (67)	12 (80)	0.350^d^
Genotype^a^					0.889^d^
A	1 (6)	2 (11)	1 (17)	0 (0)	
B	3 (18)	6 (32)	1 (17)	5 (33)	
C	4 (24)	1 (5)	1 (17)	2 (13)	
D	7 (41)	8 (42)	2 (33)	6 (40)	
E	1 (6)	2 (11)	1 (17)	2 (13)	
Unknown	1 (6)	0 (0)	0 (0)	0 (0)	
Ethnicity^a^					0.896^d^
African	5 (29)	3 (16)	1 (17)	5 (33)	
Asian	10 (59)	13 (68)	4 (67)	9 (60)	
European	2 (12)	3 (16)	1 (17)	1 (7)	
Duration (months) of HBV treatment^b^	34 (28–48)	37 (26–61)	81 (79–88)	60 (39–69)	0.042^c^
Treatment^a^					< 0.001^d^
Entecavir	13 (76)	1 (5)	1 (17)	0 (0)	
Tenofovir	4 (24)	18 (95)	5 (83)	15 (100)	
ALT (U/L)^b^	30 (24–40)	25 (19–30)	39 (18–41)	29 (21–39)	0.658^c^
Anti-HBs^b^	Negative	Negative	Negative	Negative	NA
qHBsAg (IU/mL)^b^	1607 (425–4866)	1076 (274–2320)	8464 (4739–10,255)	1393 (727–3815)	0.151^c^
HBV DNA (IU/mL)^b^	< 20	< 20	< 20	< 20	NA

Tenofovir includes both tenofovir disoproxil fumarate (n = 39) and tenofovir alanafenamide (n = 3).

*N* number of patients, *IQR* interquartile range, *HBV* hepatitis B virus, *ALT* alanine aminotransferase, *anti-HBs* antibody to hepatitis B surface antigen, *qHBsAg* quantitative hepatitis B surface antigen.

^a^Count (percentage).

^b^Median (interquartile range, IQ R).

^c^Kruskal–Wallis test.

^d^Fisher’s exact test.

More patients in the immune control group had stopped entecavir treatment whereas more patients in the viral relapse, evolving clinical relapse, and resolving clinical relapse groups had stopped tenofovir treatment (p < 0.01, Fisher’s exact test). There was also a difference in duration of antiviral treatment where patients in the evolving and resolving clinical relapse groups had a longer treatment duration before treatment cessation (p = 0.042, Kruskal–Wallis test).

**Figure 2:**
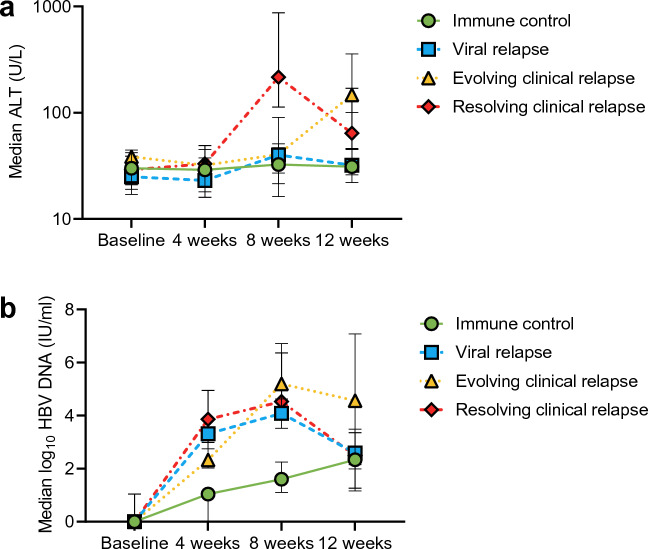
Dynamic changes in ALT and HBV DNA the initial 12 weeks after NA therapy cessation, by clinical response at 12 weeks. (**a**) Median ALT at baseline, 4 weeks, 8 weeks, and 12 weeks. (**b**) Median log_10_ HBV DNA at baseline, 4 weeks, 8 weeks, and 12 weeks. Error bars represent the interquartile ranges. *HBV* hepatitis B virus, *ALT* alanine aminotransferase.

By definition, the patients with clinical relapse, both evolving and resolving, had a higher peak ALT and HBV DNA than the other groups (Fig. 2). One of six patients in the evolving clinical relapse group and eight of 16 patients in the resolving clinical relapse group re-started treatment in week eight since they met the predefined safety criteria in the Nuc-Stop Study (ALT > 800 U/L)^19^.

### Changes in cytokines from baseline to 12 weeks

**Figure 3:**
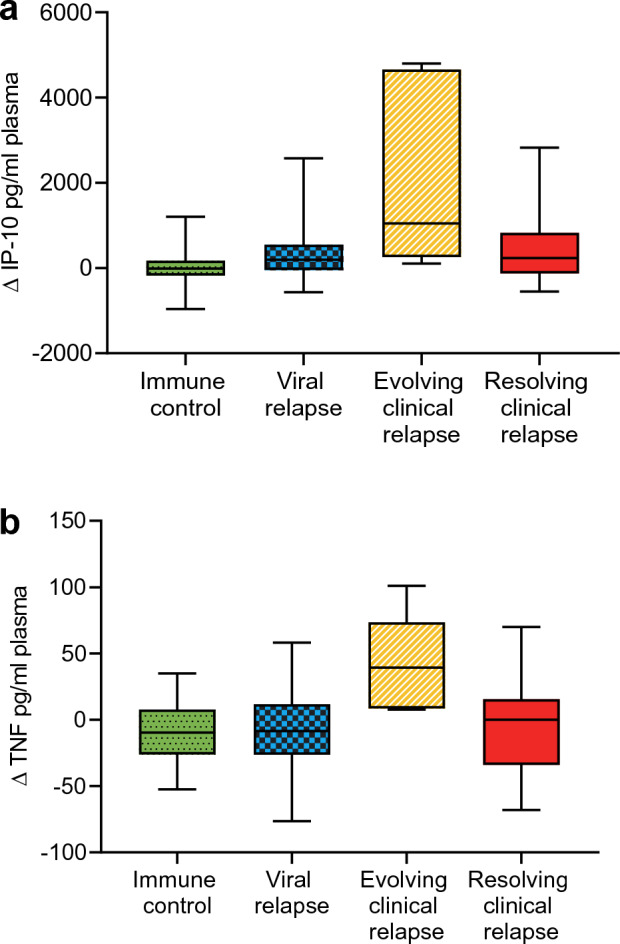
Changes in IP-10 and TNF plasma levels 12 weeks after NA therapy cessation. Shown are the differences in change (delta value between baseline and 12 weeks) in IP-10 (**a**) and TNF (**b**) from baseline to 12 weeks in the different response groups at 12 weeks. The boxes represent the interquartile ranges (IQR), and the middle lines represent the medians. *IP-10* interferon-γ-induced protein 10, *TNF* tumor necrosis factor.

**Figure 4:**
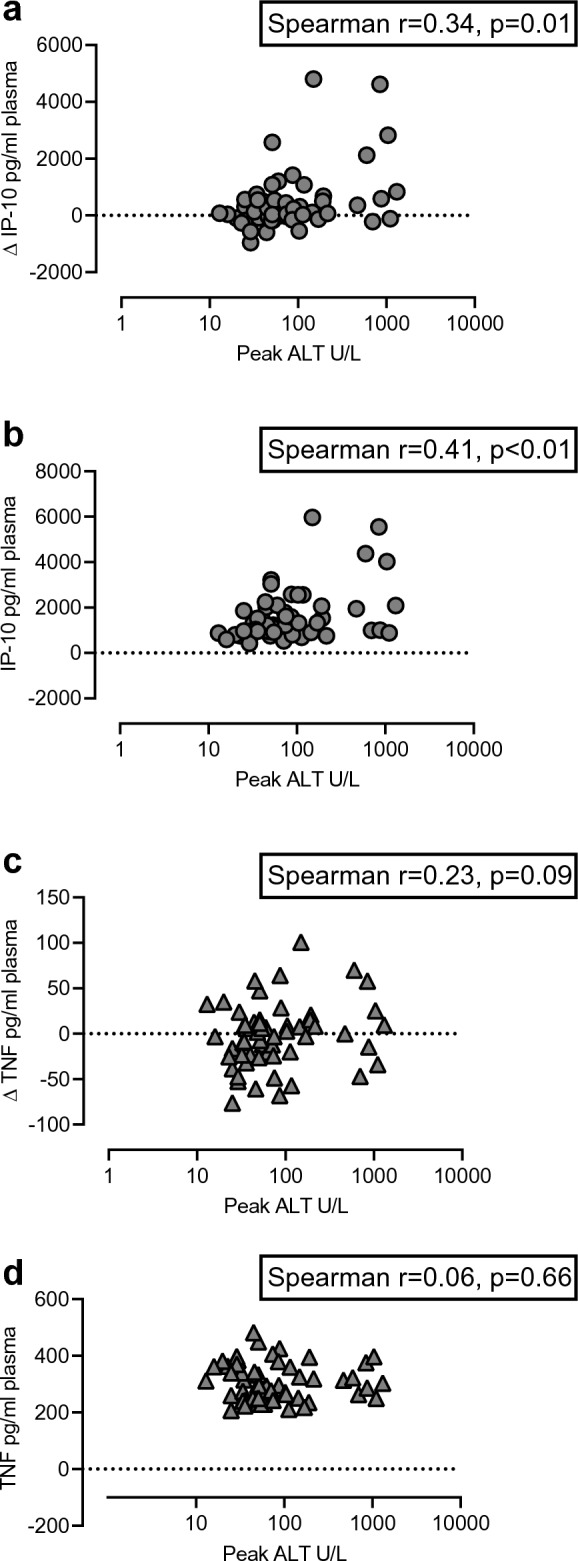
Scatter diagrams of peak ALT and change in IP-10 and absolute IP-10 at 12 weeks, and peak ALT and change in TNF and absolute TNF at 12 weeks. (**a**) Correlation between peak ALT and change in IP-10. (**b**) Correlation between peak ALT and absolute IP-10 at 12 weeks. (**c**) Correlation between peak ALT and change in TNF. (**d**) Correlation between peak ALT and absolute TNF at 12 weeks. The values for Spearman’s correlation coefficient are given. Peak ALT is the highest ALT value measured during the first 12 weeks after NA therapy cessation. Change in IP-10 and TNF are the delta values for IP-10 and TNF between baseline and 12 weeks. Absolute IP-10 and TNF are the values for IP-10 and TNF measured at 12 weeks after NA therapy cessation. *ALT* alanine aminotransferase, *IP-10* interferon-γ-induced protein 10, *TNF* tumor necrosis factor.

The change in cytokine concentrations from baseline to 12 weeks was calculated, and the four clinical response groups: immune control, viral relapse, evolving clinical relapse, and resolving clinical relapse, were compared. There was a significant difference in change in IP-10 between the four groups (p = 0.012, Kruskal–Wallis test) (Fig. 3). Patients with evolving clinical relapse had a significantly greater increase in IP-10 compared with the immune control group (p = 0.007, Bonferroni post-hoc test) but not compared with the other groups. There was a weak correlation between peak ALT and change in IP-10 (Spearman r = 0.34, p = 0.01) (Fig. 4a). The correlation between peak ALT and absolute IP-10 at 12 weeks was stronger (Spearman r = 0.41, p < 0.01) (Fig. 4b).

Between the four groups, there was also a significant difference in change of TNF from baseline to 12 weeks (p = 0.032, Kruskal–Wallis test) (Fig. 3) with a significantly greater increase in evolving clinical relapse compared with the immune control (p = 0.036, Bonferroni post-hoc test) and viral relapse (p = 0.031, Bonferroni post-hoc test) groups. Peak ALT did not correlate with change in TNF (Spearman r = 0.23, p = 0.09) (Fig. 4c). Likewise, there was no correlation between peak ALT and absolute TNF at 12 weeks (Spearman r = -0.06, p = 0.66) (Fig. 4d).

There were no significant differences between the four clinical response groups for changes in any of the other 20 cytokines.

**Figure 5:**
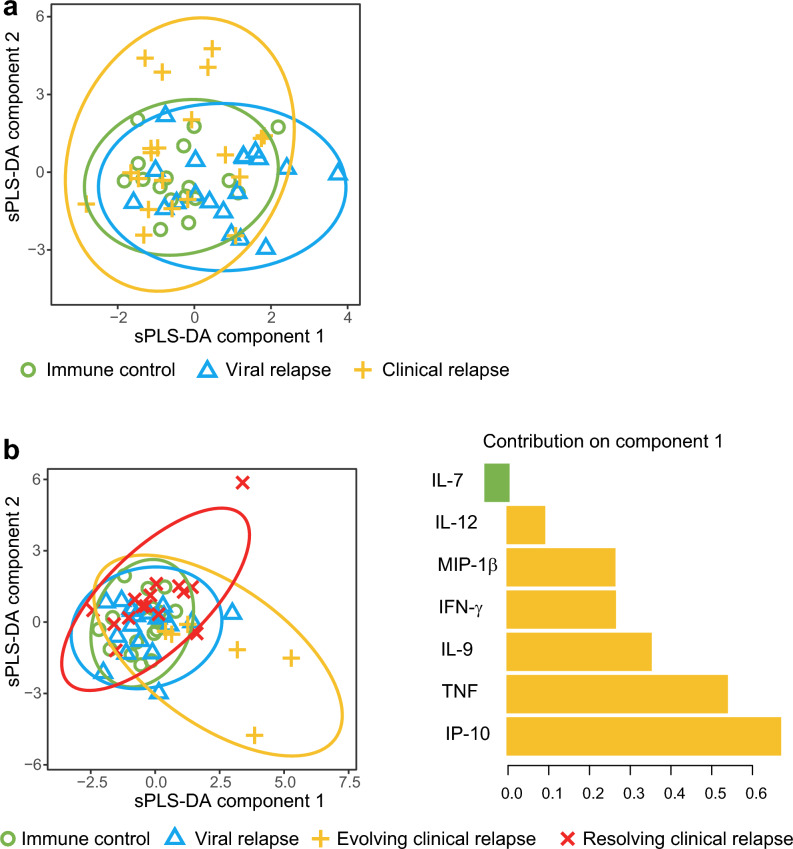
Sparse partial least square discriminant analysis (sPLS-DA) of baseline cytokines and changes in cytokines 12 weeks after NA therapy cessation. (**a**) Two-dimensional sPLS-DA of baseline cytokines in the immune control, viral relapse, and clinical relapse groups. (**b**) Two-dimensional sPLS-DA of changes in plasma levels of cytokines from baseline to 12 weeks (delta values) in the immune control, viral relapse, evolving clinical relapse, and resolving clinical relapse groups. To the right are the main contributing variables to component 1 for the delta values. IP-10, TNF, IL-9, IFN-γ, MIP-1β, and IL-12 are the main drivers for a more positive first component in the evolving clinical relapse group. *sPLS-DA* sparse Partial Least Square Discriminant Analysis, *IP-10* IFN-γ-induced protein 10, *TNF* tumor necrosis factor, *IL-9* interleukin-9, *IFN-γ* interferon-γ, *IL-12* interleukin-12.

When looking at the change in cytokines from baseline to 12 weeks using sPLS-DA, the evolving clinical relapse group had higher first component values than the immune control, viral relapse, and resolving clinical relapse groups. This separation was driven mainly by IP-10, TNF, IL-9, IFN-γ, MIP-1β, and IL-12 (Fig. 5b).

### Cytokine analysis at baseline

**Figure 6:**
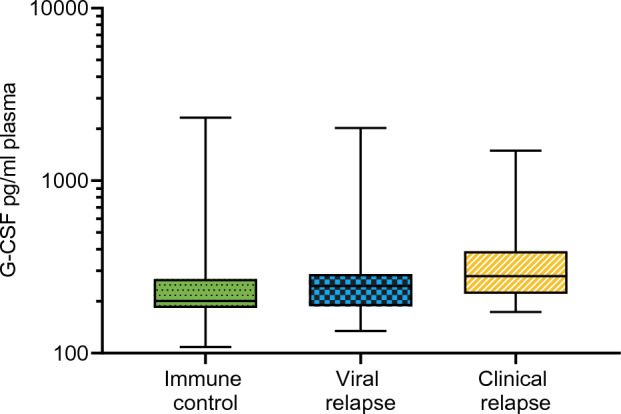
Baseline G-CSF before NA therapy cessation. Shown are the baseline values of C-CSF in the different clinical response groups at 12 weeks. The boxes represent the interquartile ranges (IQR), and the middle lines represent the medians. *G-CSF* granulocyte colony-stimulating factor.

The differences in baseline cytokine concentrations between patients with immune control, viral relapse, and clinical relapse at 12 weeks were assessed. As displayed in Fig. 6, we found a significantly higher G-CSF value at baseline for patients with clinical relapse (p = 0.043). Using the Bonferroni post hoc test, G-CSF was significantly higher only when comparing patients with clinical relapse to the immune control group (p = 0.039).

There were no significant differences between the clinical response groups in any of the other 21 cytokines.

A ROC analysis was performed to define an optimal cut-off for baseline G-CSF to discriminate between patients who experienced clinical relapse and not. The cut-off that maximized specificity and sensitivity was 250 pg/ml, with a sensitivity of 0.71, specificity of 0.64, positive predictive value of 0.54, negative predictive value of 0.79 and AUC of 0.68.

As an unbiased approach, sPLS-DA analysis was performed to investigate whether integrated cytokine profiles at baseline could predict clinical and virological response 12 weeks after treatment cessation. There was no clear separation of the response groups (immune control, viral relapse, and clinical relapse) based on the 22 baseline cytokines included in the analysis (Fig. 5a).

## Discussion

Cytokines are an indispensable part of the immune response, and several studies have investigated the role of cytokines in HBV infection^14,22–25^. However, the role the individual cytokines play in HBV infection pathogenesis and disease progression is poorly understood^25^. In this study, we found that evolving clinical relapse 12 weeks after NA cessation was associated with specific increases in IP-10 and TNF. The separation of the evolving clinical relapse group from the other three response groups at 12 weeks (immune control, viral relapse, and resolving clinical relapse), was visualized by sPLS-DA and driven mainly by IP-10, TNF, IL-9, IFN-γ, and IL-12.

IP-10, also known as C-X-C motif chemokine ligand-10 (CXCL10), is secreted by a diversity of cells, including but not limited to monocytes, neutrophils, eosinophils, activated T lymphocytes, natural killer cells, endothelial cells, and hepatocytes^26,27^. It is a chemokine (chemotactic cytokine) that attracts peripheral leucocytes into the liver^25^. In acute HBV infection, IP-10 is found to be higher in patients who resolve the infection than in patients who develop persistent infection^22^. We found a significant increase in IP-10 plasma levels, 12 weeks after treatment cessation in patients with evolving clinical relapse (viz a progressive flare). This is consistent with previous studies that have found an increase in IP-10 during hepatic flares^24,28^. It has been argued that IP-10 can be used as a predictor of treatment response and progression of disease^25^. Wang et al. found that IP-10 was an independent predictor of significant fibrosis, and that the serum level of IP-10 was positively correlated with the severity of liver fibrosis^26^. Serum IP-10 level has also been found to be associated with HBsAg seroclearance in CHB^29^. IP-10 probably plays a key role in HBV infection and may be useful in distinguishing the phases of infection, with the highest levels in active hepatitis^30^. Thus, our finding is consistent with prior observations and confirms the importance of IP-10 in the immune response against HBV.

TNF is a proinflammatory cytokine mainly produced by monocytes and macrophages. It has been shown in vitro that human macrophages secrete TNF in response to high titres of HBV^31^. Among CHB patients with liver cirrhosis, TNF is found to correlate with ongoing inflammation^32^. An association between liver damage and TNF producing CD4 T-cells has been found in patients with CHB flare^33^. TNF plays an important role in the defence against many infections, and the role of TNF in HBV immunity is supported by the finding that the use of TNF inhibitors can lead to HBV reactivation^34^. Our finding of an elevation in TNF in patients with evolving clinical relapse is in line with a study by Hall et al., who found increased activity of TNF in patients with hepatitis flares after stopping NA therapy^24^. Another study found no increase in TNF in patients with a CHB flare^28^, but this study included only five patients stopping NA therapy. Our observation of an increase in TNF during a HBV flare is plausible, as TNF is a potent proinflammatory cytokine.

In line with our findings from the conventional statistical analyses, sPLS-DA showed a higher first component value for the evolving clinical relapse group compared to the immune control, viral relapse, and resolving clinical relapse groups when looking at the change in cytokines from baseline to 12 weeks. This separation was driven mainly by IP-10, TNF, IL-9, IFN-γ, MIP-1β, and IL-12, all pro-inflammatory cytokines, suggesting that these are upregulated during a progressive flare. Both IFN-γ and MIP-1β have been found to increase after CHB treatment cessation^35^. IL-12 levels are associated with ALT levels, and high levels of IL-12 has also been linked to HBeAg or HBsAg seroconversion^25,36^.

We found that G-CSF at baseline was elevated in patients with clinical relapse 12 weeks after NA therapy cessation. G-CSF is a hematopoietic growth factor and stimulates neutrophil development and differentiation^37^. G-CSF therapy has been used in HBV associated acute on chronic liver failure, and some Asian studies have found improved liver function and survival rates in patients receiving this treatment^38,39^. However, a European multicentre study on acute on chronic liver failure of different etiologies failed to demonstrate any positive effect of G-CSF therapy^40^. To our knowledge, the role of G-CSF in HBV infection is still elusive, and further exploration is warranted.

At present, baseline cytokines have not proven their role as predictors of outcome after treatment cessation in CHB. Wübbolding et al. have suggested that a combination of cytokines can be valuable as predictors of outcome but found poor performance of single cytokines^23^. Hall et al. found no difference in baseline cytokine production between flare and non-flare patients with HBeAg-negative CHB who stopped NA treatment, but their analyses did not include G-CSF^24^. Our finding that sPLS-DA analysis showed no clear differentiation of baseline cytokine profiles between the 12-weeks clinical response groups are in line with these observations. The ROC analysis we performed to further examine the ability of baseline G-CSF to predict outcome yielded a low AUC of 0.68, indicating that baseline G-CSF alone is not suitable for this purpose. Our established cut-off of 250 pg/ml had limited value in clinical decision making due to insufficient sensitivity and specificity.

After stopping NA therapy, about 20–25% of patients experience an ALT flare that typically occurs during the first 12–24 weeks after treatment cessation^41^. It has been established that stopping tenofovir leads to an earlier flare than stopping entecavir; usually patients stopping tenofovir experience a flare within 12 weeks, whereas patients stopping entecavir experience a flare closer to 24 weeks or later^42–45^. The mechanisms behind the difference in relapse patterns are unclear, but may be due to differences in immunomodulatory effects^46^. We measured cytokines at baseline and 12 weeks after NA cessation, and type of NA therapy could have affected our results. As expected, most patients treated with entecavir were in the immune control group whereas most patients treated with tenofovir were in the viral and clinical relapse groups at 12 weeks (Table 1).

Re-start of antiviral treatment was more common in the resolving clinical relapse group, where eight patients re-started treatment at the 8 weeks visit, compared to the evolving clinical relapse group, where only one patient re-started treatment at week eight. One could argue that re-treatment at week eight led to a fall in HBV DNA and ALT and consequently classification as resolving clinical relapse four weeks later. Thus, re-treatment may have impeded an immunological process in these patients.

Duration of NA treatment before cessation differed significantly across the clinical response groups; the longest median duration was observed in the evolving clinical relapse group (81 months) and the shortest in the immune control group (34 months). Duration of NA treatment is perceived as vital for the outcome in stop-studies as longer duration of viral suppression is thought to be associated with enhanced immune restoration^17^. Our study supports this hypothesis as the evolving clinical relapse group with presumably the highest immune activity was the group with the longest treatment prior to cessation.

The strength of our study was the rigorous, prospective study design where all patients underwent close monitoring after treatment cessation. All common viral genotypes were represented, as well as different age groups and ethnicities.

The study also had some limitations. First, the sample size was small. In particular, the small size of the evolving clinical relapse group limited statistical power, and there are possibly other interesting cytokine dynamics that we failed to unravel. Second, we only measured cytokine levels at baseline and 12 weeks thereafter. Due to the short half-lives of cytokines, we may have missed possibly intricate dynamics. Finally, we only described short-term response (12 weeks), and longer follow-up is needed to observe if these early changes in cytokines and chemokines can predict HBsAg loss which usually happens several months to years after NA cessation. We will continue to follow these patients to evaluate if the cytokine dynamics between baseline and 12 weeks of follow-up can predict long-term effects of treatment cessation.

In conclusion, we found that the immunological response occurring with HBV reactivation after NA cessation is associated with an increase in IP-10 and TNF. Longer follow-up is needed to evaluate whether these cytokines can distinguish beneficial flare from detrimental flare and predict long-time outcome after treatment withdrawal in chronic HBeAg-negative hepatitis B.

## Data Availability

The data that support the findings of this study are available upon reasonable request from the corresponding author.
